# Minor physical anomalies and social cognition in patients with schizophrenia and their first degree relatives

**DOI:** 10.3389/fpsyt.2025.1679635

**Published:** 2025-11-06

**Authors:** Safa Güney, Mine Sahingoz, Esat Fahri Aydın

**Affiliations:** 1 Ataturk Universitesi Tip Fakultesi, Erzurum, Türkiye; 2 Necmettin Erbakan Universitesi Tip Fakultesi, Karatay, Türkiye

**Keywords:** schizophrenia, minor physical anomalies, dysmorphogenesis, social cognition, theory of mind (ToM)

## Abstract

**Objective:**

Minor physical anomlalies (MPAs) are early dysmorphogenetic findings that can be frequently detected in both schizophrenia patients and their relatives, and deficits in social cognitive abilities have been shown in both groups. We aimed to investigate whether there is a relationship between MPAs and social cognition in both the patient group and the patient relatives.

**Method:**

Thirty-four schizophrenia patients in remission, 34 their first-degree relatives, and 34 healthy controls were included in the study. Participants were assessed for minor physical anomalies using the Minor Physical Anomalies Scale (MPAS), and for theory of mind using Reading the Mind in the Eyes Test (RMET) and Dokuz Eylül Theory of Mind Index (DEToMI).

**Results:**

Patients performed significantly worse than healthy controls on the DEToMI subtests ‘first-order false belief’, ‘second-order false belief’, ‘faux pas’ and ‘DEToMI total’ (p=0.003, p=0.001, p=0.016 and p=0.001, respectively). The mean scores of patients in RMET were significantly lower than those of patients’ relatives and healthy controls (p=0.012 and p<0.001, respectively). Patients with mouth anomalies performed worse on the first-order false belief subtest than those without (p=0.029), and there was a negative correlation between mouth involvement and first-order false belief subtest performance in group I (r=-0.381; p=0.026). Patients with ear anomalies performed better on the irony/hinting subtest than those without (p=0.048), and there was a positive correlation between ear involvement and irony/hinting subtest performance in group I (r=0.364; p=0.034). Relatives of patients with mouth anomalies performed worse than those without on the second-order false belief subtest (p=0.043), and there was a negative correlation between mouth involvement and second-order false belief subtest performance in group II (r=-0.353; p=0.041). Apart from these significant findings, no significant association was found between DEToMI and RMET performances and MPAs in all groups.

**Conclusion:**

The present study’s findings considering the associations between minor physical anomalies and social cognition in schizophrenia patients and their first degree relatives would supply new perspectives in the clinicians’ assessment of schizophrenia patients. Further research of this possible link with similar studies may be beneficial in better understanding the nature of the disease and in more comprehensive clinical evaluations of patients.

## 1 Introduction

Schizophrenia is a complex psychiatric disorder that is presented by positive symptoms, negative symptoms and cognitive impairment. The cognitive deficits seen in schizophrenia have a special importance in the course of the disorder due to the fact that they do not benefit enough from antipsychotic treatments, and seem to be especially related to long-term psychosocial functioning of patients with schizophrenia (1–3). These cognitive deficits can be found in the premorbid period of the patients and in the relatives of the patients, albeit at milder levels (3).

Cognition can be divided into neurocognition and social cognition, and patients with schizophrenia spectrum disorders may show deficits at various levels in both neurocognition and social cognition (4). However, it was stated that deficits in social cognition in schizophrenia are more effective on functionality of the patients than those in neurocognition (3). Social cognition is defined as representing the relationship between oneself and others and processing this representation with social behaviors (5). Social cognition is divided into five subdomains: theory of mind, social perception, social knowledge, attributional bias, and emotional processing (6). Theory of mind (ToM) is conceptualized as the ability to understand that others have mental states different from one’s own and to infer the mental states of others, including their intentions and beliefs (7), it explains social cognition well, has been studied extensively in recent years, and has been shown to be impaired in patients with schizophrenia (8–10). The ability to understand the mental state of others by the help of ToM may be the best cognitive mediator of social functioning in schizophrenia (11). ToM deficits in schizophrenia have been consistently associated with certain areas of the brain, suggesting a certain biological background (11). Deficits of ToM was found in the relatives of the patients with schizophrenia, and impairments of ToM may be a candidate for an endophenotype in schizophrenia (12).

Minor physical anomalies (MPAs) are morphological variations that do not cause significant cosmetic problems. MPAs are based on the fetal neurodevelopmental period (13). These anomalies, which originate from the ectoderm layer where the major development of the brain takes place and share their embryonic substrates with the brain, are thought to be markers of developmental disorders in the central nervous system (CNS) of the fetus (13–15). This assumption coincides with the hypothesis that schizophrenia is a neurodevelopmental disorder (14). Previously, it has been found that these anomalies were found at a higher rate in patients with schziophrenia than in healthy controls (14, 16). Additionally, these anomalies can be seen at a higher rate in schizophrenia patients’ relatives compared to healthy controls (17, 18). Although the role of genetic and environmental factors in the etiology of MPAs is unclear, these anomalies appear to predict clinical features of schizophrenia, such as earlier age of onset and a more severe course (14, 19). As we mentioned above, considering the parallel morphogenetic processes between minor physical anomalies and the brain, it can be assumable that there may be a connection between MPAs and ToM ability, in patients with schizophrenia. To clarify these possible links mentioned above, we aimed to investigate the relationship between ToM and MPAs in schizophrenia patients, their first-degree relatives, and healthy controls. Our hypotheses were as follows: 1) minor physical anomalies would be seen at the highest rate in the schizophrenia patient group, at the lowest rate in the healthy control group, and between these two groups in the patient’s relatives group, 2) ToM performance would be worst in the schizophrenia patient group, best in the healthy control group, and between these two groups in the patient’s relative group, 3) increased MPA rates in schizophrenia patients and first-degree relatives of schizophrenia patients would be associated with increasing impairment in ToM.

## 2 Materials and methods

### 2.1 Setting and sample

The study included 34 schizophrenia patients (Group I) who consecutively applied to the Atatürk University Faculty of Medicine, Department of Psychiatry outpatient clinic between June 2019 and March 2021, were diagnosed with schizophrenia according to Diagnostic and Statistical Manual of Mental Disorders-5 (20), and were in remission for at least 8 weeks. Additionally, 34 first-degree relatives of schizophrenia patients (Group II) and 34 healthy controls (Group III) were included in the study. All three groups were matched for age and education levels. The inclusion criteria for the participants were as follows: being literate, between the ages of 18-65, diagnosed with schizophrenia with SCID-I (21). The exclusion criterion for patients was the presence of any additional psychiatric disorder according to the interview with SCID-I. The presence of any psychiatric disorder and/or organic mental disorder (e.g., dementia) according to the interview with SCID-I in the patient’s relatives and healthy controls was also an exclusion criterion.

### 2.2 Procedures and materials

Approval for our study was obtained from the local ethics committee of Atatürk University Faculty of Medicine, dated 26.12.2019 and decision number 25. The participants included in the study were informed about the purpose of the study, the physical examination and psychometric tests to be applied by the first author of the study, and their written consent was obtained. A sociodemographic-clinical data form prepared by the researchers considering the aims of the study was applied to the partcipants.

In our study, Positive and Negative Syndrom Scale (PANSS) was applied to schizophrenia patients. In addition, to all participants; Dokuz Eylül Theory of Mind Index (DEToMI) and Reading the Mind in the Eyes Test (RMET) were applied to evaluate ToM abilities, and the MPA scale (MPAS) was applied to evaluate MPAs.

PANSS (22) is a thirty-item semi-structured scale developed to assess positive (P) symptoms (7 items), negative (N) symptoms (7 items), and general psychopathology (G) (16 items) in patients with schizophrenia or other psychotic disorders; scoring ranges from 1 (no abnormality) to 7 (very severe). Turkish validity and reliability study of the scale was established (23). In our study, the remission period in Lieberman’s study (24) and PANSS scoring in Andreasen’s study (25) were used as remission criteria in schizophrenia. Accordingly, remission is a score of 3 or less from delusions (P1), conceptual disorganization (P2), hallucinatory behaviour (P3), blunted affect (N1), passive-apethetic social withdrawal (N4), lack of spontaneity and flow of conversation (N6), mannerism and posturing (G5), unusual thought content (G9) items on PANSS scale for at least eight weeks.

Dokuz Eylül Theory of Mind Index (DEToMI) (26) includes four questions assesing first-order false belief (the ability to understand another person’s beliefs), three questions assesing second-order false belief (the ability to understand a second person’s thoughts about a third person’s thoughts), three questions assesing irony/hinting, two questions assesing metaphor, three questions assesing empathic understanding, and one question assesing faux pas (to say something or do something without considering whether it might hurt the other person). The scale, which includes two pictures and seven story tasks, consists of a total of 16 questions. Scoring was made as “1” for correct answer and “0” for incorrect answer, through an answer key containing predetermined correct answers. Higher scores mean better ToM abilities. A validity and reliability study was conducted in patients with schizophrenia (27). RMET (28) was designed by Baron-Cohen et al. in 1997 to include 25 questions to measure social cognitive abilities (29). In the RMET, it is aimed to analyze the mental state of the person from facial expressions. Turkish validity and reliability study of the test was conducted by Yıldırım et al. in 2011 and it consists of 32 questions (30). In the test, for each pair of eyes, the word that best describes what the person in the picture is thinking or feeling is asked to choose from among the four options. Each correct answer is given one point. A high score on the scale means good theory of mind abilities.

In the MPA scale developed by Yoshitsugu et al. (31); head circumference, epicanthus, hypertelorism (distance between tear ducts), low-seated ear, adherent ear-lobe, asymmetrical ears, high palate, furrowed tongue, curved fifth finger (angling of the little finger), single transverse palmar crease, long third toe, strabismus and cuspidal ear features are evaluated.

### 2.3 Statistical analysis

Statistical analysis of our study was performed with the Statistical Package for the Social Sciences (SPSS) 23.0 program. In descriptive statistics, number (n) and percentage (%) were used for categorical data, mean (Mean) and standard deviation (s.s.) were used for numerical data. Chi-square was used in the comparison of categorical data between both groups, and Fisher-Exact test was used in cases where the assumptions were not met. ANOVA and Student’s t test were used to compare the normally distributed numerical data, and the Kruskal Wallis and Mann-Whitney U tests were used to compare the non-normally distributed numerical data. Pairwise comparisons adjusted using Tukey test for normally distributed continuous variables and Bonferroni correction for non-normally distributed continuous variables and categorical variables. Pearson test was used for the correlations between the numerical data that fit the normal distribution, and the Spearman’s test was used for the correlations between the numerical data that did not fit the normal distribution. Statistical significance level was accepted as p<0.05.

## 3 Results

### 3.1 Sample characteristics

A total of 102 individuals, 34 of whom were schizophrenic patients (Group I), 34 first-degree relatives of patients with schizophrenia (Group II), and 34 healthy controls (Group III) were included in the study. The demographic characteristics of the sample are shown in **Table 1**. The average age for groups I, II and III were 34.67 (± 9.77), 38.41 (± 11.11), 38.35 (± 10.88) years, respectively. The average years of education levels for groups I, II and III were 11.47 (± 3.52), 11.75 (± 4.03) and 12.08 (± 3.85), respectively. There was no significant difference between the three groups in terms of age and education levels (p<0.05). There were 15 female participants and 19 male participants in group I. The numbers of female and male participants in each of group II and group III were 6 and 28, respectively.

**Table 1:** Basic demographic characteristics of the three groups.

		Group I	Group II	Group III	p
		mean ± SD/n-%	mean ± SD/n-%	mean ± SD/n-%	
Age(year)		34.67 ± 9.77	38.41 ± 11.11	38.35 ± 10.88	.255^A^
Gender	Female	15-%44.1	6-%17.6	6-%17.6	**.017** ^χ2^
	Male	19-%55.9	28-%82.4	28-%82.4	
Education (year)		11.47 ± 3.52	11.75 ± 4.03	12.08 ± 3.85	.800^A^

^A^ANOVA and ^χ2^ Chi-squared test. Significant outcomes of P-values are reported in bold.

The mean PANSS scores of the patient group: Positive symptoms subscale 9.88 (± 3.6), negative symptoms subscale 12.82 (± 4.4), general psychopathology subscale 23.17 (± 4.49), total 45.88 (± 10.1) was found. All patients were using antipsychotic medication, and it was determined that 2.7% used first-generation antipsychotic (FGA) and 97.3% used second-generation antipsychotic (SGA).

### 3.2 Comparison of minor physical anomalies between the groups

Minor physical anomalies were classified in four main anatomical regions: mouth, eye, ear and limb (**Table 2**).

**Table 2:** Comparison of minor physical anomalies between the groups-1.

		Group I		Group II		Group III		p^χ 2^
		n	%	n	%	n	%	
Eye	(-)	34	100%	34	100	33	97.1%	(-)
	(+)	0	0%	0	0%	1	2.9%	
	Epicanthus	0	0%	0	0%	0	0%	
	Strabismus	0	0%	0	0%	1	2.9%	(-)
Ear	(-)	21	61.8%	28	82.4%	25	73.5%	.162
	(+)	13	38.2%	6	17.6%	9	26.5%	
	Low seated ear	1	3.1%	0	0%	0	0%	(-)
	Adherent ear lobe	9	26.5%	5	14.7%	3	8.8%	.139
	Assymetrical ears	4	11.8%	3	8.8%	6	17.6%	(-)
	Cuspidal ear	0	0%	0	0%	0	0%	
Mouth	(-)	21	61.8%	27	79.4%	27	79.4%	.163
	(+)	13	38.2%	7	20.6%	7	20.6%	
	High palate	4	12.1%	1	2.9%	1	2.9%	(-)
	Furrowed tongue	11	32.4%	6	17.6%	6	17.6%	.246
Limb	(-)	20	58.8%	17	50%	23	67.6%	.335
	(+)	14	41.2%	17	50%	11	32.4%	
	Curved fifth finger	14	41.2%	17	50%	9	26.5%	.133
	Single transverse palmar crease	0	0%	0	0%	1	2.9%	(-)
	Long third toe	1	2.9%	0	0%	1	2.9%	(-)

^χ2^ Chi-square test.

There was no eye anomaly in groups I (schizophrenia patients) and II (patient’s relatives). One person (2.9%) in group III (healty controls) had strabismus. In the comparison of the eye involvement of the groups, the p value could not be calculated because sufficient number of eye anomalies were not detected (**Table 2**).

Ear involvement was present in 13 subjects (38.9%) in Group I, 6 subjects (17.6%) in Group II, and 9 subjects (26.5%) in Group III, and there was no significant difference between the groups (p= 0.162). Among all participants, only one person in Group I had a low-seated ear. Adherent ear-lobe was detected in 13 subjects (38.2%) in group I, 5 subjects (14.7%) in group II, and 3 subjects (8.8%) in group III, and considering the presence of adherent ear-lobe in the three groups there was no significant difference between the groups (p=0.139). Asymetrical ears were detected in 4 subjects (11.8%) in group I, 3 subjects (8.8%) in group II, and 6 subjects (17.6%) in group III. The p value could not be calculated due to the low number of asymmetrical ears. No cuspidal ear was detected in any group (**Table 2**).

Mouth involvement was present in 13 subjects (38.2%) in group I and in 7 subjects (20.6%) in equal in both group II and III, and there was no significant difference between the groups (p=0.163). High palate was detected in 4 subjects (12.1%) in group I and in one subject (2.9%) in equal in both group II and III, and the p value could not be calculated due to low number of high palate. Furrowed tongue was detected in 11 subjects (32.4%) in group I and in 6 subjects (17.6%) in equal in both groups II and III, and there was no significant difference between the groups (p=0.246) (**Table 2**).

Limb involvement was present in 14 subjects (41.2%) in group I, 17 subjects (50%) in group II, and 11 subjects (32.4%) in group III, and there was no significant difference between the groups (p=0.335). Curved fifth finger was present in 14 subjects (41.2%) in group I, 17 subjects (50%) in group II, and 9 subjects (26.5%) in group III, and there was no significant difference between the groups (p=0.133). Single transverse palmar crease was detected in only one (2.9%) subject in group III among all groups, and the p value could not be calculated because the number of palmar line anomalies was low. Toe (long third toe) anomaly was detected in one subject (2.9%) in both groups I and III, and p value could not be calculated (**Table 2**).

Mean head circumference was 56.25 (± 2.27) cm in group I, 57.08 (± 1.79) cm in group II, 56.95 (± 2.12) cm in group III. Mean distance between tear ducts (hypertelorism) were 29.20 (± 3.48) mm in group I, 28.29 (± 2.83) mm in group II, 28.91 (± 2.65) mm in group III. There was no significant difference between the 3 groups in terms of each of mean head circumference (cm), hypertelorism (mm) (**Table 3**).

**Table 3:** Comparison of minor physical anomalies between the groups-2.

	Group I	Group II	Group III	p
	mean ± SD	mean ± SD	mean ± SD	
Head circumference (cm)	56.25 ± 2.27	57.08 ± 1.79	56.95 ± 2.12	.211^A^
Hypertelorism (mm)	29.20 ± 3.48	28.29 ± 2.83	28.91 ± 2.65	.423^K^

^A^ANOVA and ^K^Kruskal-wallis test. Significant outcomes of P-values are reported in bold.

### 3.3 Comparison of DEToMI and RMET scores between the groups

In ‘first-order false belief’, ‘second-order false belief’, ‘faux pas’ and ‘DEToMI total’, group III received the highest average score, group I received the lowest average score, the average scores of group II were between these two groups; and the differences between the three groups were significant (p=0.011, p=0.003, p=0.043, p=0.002 respectively) (**Table 4**). When the groups were compared pairwise among themselves, significant differences were found only between group I and group III in terms of the mean scores of ‘first-order false belief’ (p = 0.003), ‘second-order false belief’ (p = 0.001), ‘faux pas’ (p = 0.016) and ‘DEToMI total’ (p = 0.001).

**Table 4:** Comparison of DEToMI scores between the groups.

	Group I (n=34)	Group II (n=34)	Group III (n=34)	P
	mean ± SD	mean ± SD	mean ± SD	
First order false belief	2.67 ± 0.91	2.76 ± 1.28	3.32 ± 1.03	**.011^K^**
Second order false belief	1.32 ± 0.87	1.85 ± 0.98	2.08 ± 0.79	**.003^K^**
Irony/hinting	1.44 ± 1.10	1.38 ± 1.25	1.76 ± 1.07	.343^K^
Metaphor	1.00 ± 0.77	1.20 ± 0.72	1.29 ± 0.75	.260^K^
Empathic understanding	2.52 ± 0.82	2.82 ± 0.45	2.88 ± 0.32	.094^K^
Faux pas	0.14 ± 0.35	0.23 ± 0.43	0.41 ± 0.49	**.043^K^**
Total score	9.17 ± 2.71	10.23 ± 3.42	11.76 ± 2.45	**.002^A^**

^A^ANOVA and ^K^Kruskal-wallis test. Significant outcomes of P-values are reported in bold.

In RMET, group III received the highest average score, group I received the lowest average score, the average scores of group II were between these two groups; and the differences between the three groups were significant (p<0.001) (**Table 5**). When the groups were compared among themselves in pairs in terms of RMET, the mean score of group I was significantly lower than group II and group III (p=0.012 and p<0.001, respectively). Group II scored lower than group III, but the difference was not significant (p = 0.228).

**Table 5:** Comparison of RMET scores between the groups.

	Group I (n=34)	Group II (n=34)	Group III (n=34)	p
	mean ± SD	mean ± SD	mean ± SD	
RMET score	17.35 ± 5.78	21.02 ± 5.27	23.11 ± 4.46	**.000^A^**

^A^ANOVA. Significant outcomes of P-values are reported in bold. RMET, Reading the Mind in the Eyes Test.

### 3.4 Comparison of minor physical anomalies with DEToMI and RMET scores

When those with and without mouth involvement in group I were compared in terms of theory of mind tests, only in DEToMI first-order false belief subtest, the mean test score of the group with mouth involvement was significantly lower than the group without mouth involvement (p=0.029) (**Table 6**).

**Table 6:** Comparison of mouth anomaly presence with ToM tests in group I.

Group I	Mouth anomaly (-)	Mouth anomaly (+)	p
	mean ± SD	mean ± SD	
RMET	18.47 ± 6.02	15.53 ± 5.07	.153^t^
First order false belief	2.95 ± 0.86	2.23 ± 0.83	**.029** ^m^
Second order false belief	1.47 ± 0.74	1.07 ± 1.03	.222^m^
Irony/hinting	1.38 ± 1.07	1.53 ± 1.19	.700^m^
Metaphor	0.95 ± 0.8	1.07 ± 0.75	.650^m^
Empathic understanding	2.61 ± 0.8	2.38 ± 0.86	.354^m^
Faux pas	0.19 ± 0.4	0.07 ± 0.27	.371^m^
DEToMI total score	9.50 ± 2.58	8.53 ± 2.9	.288^t^

^m^Mann–Whitney’s U-test and ^t^ T-test. Significant outcomes of P-values are reported in bold. DEToMI, Dokuz Eylül Theory of Mind Index; RMET, Reading the Mind in the Eyes Test.

When those with and without ear involvement in group I were compared in terms of theory of mind tests, only in DEToMI irony/hinting subtest, the mean test score of the group with ear involvement was significantly higher than the group without ear involvement (p=0.048) (**Table 7**).

**Table 7:** Comparison of ear anomaly presence with ToM tests in group I.

Group I	Ear anomaly (-)	Ear anomaly (+)	p
	mean ± SD	mean ± SD	
RMET	16.52 ± 6.27	18.69 ± 4.81	.295^t^
First order false belief	2.47 ± 0.92	3.00 ± 0.81	.093^m^
Second order false belief	1.28 ± 0.95	1.38 ± 0.76	.778^m^
Irony/hinting	1.14 ± 0.96	1.92 ± 1.18	**.048** ^m^
Metaphor	0.85 ± 0.72	1.23 ± 0.83	.174^m^
Empathic understanding	2.61 ± 0.74	2.38 ± 0.96	.401^m^
Faux pas	0.14 ± 0.35	0.15 ± 0.37	.931^m^
DEToMI total score	8.61 ± 2.85	10.07 ± 2.28	.130^t^

^m^Mann–Whitney’s U-test and ^t^ T-test. Significant outcomes of P-values are reported in bold. DEToMI, Dokuz Eylül Theory of Mind Index; RMET, Reading the Mind in the Eyes Test.

When those with and without mouth involvement in group II were compared in terms of theory of mind tests, only in DEToMI second-order false belief subtest, the mean test score of the group with mouth involvement was significantly lower than the group without mouth involvement (p=0.043) (**Table 8**).

**Table 8:** Comparison of mouth anomaly presence with ToM tests in group II.

Group II	Mouth anomaly (-)	Mouth anomaly (+)	p
	mean ± SD	mean ± SD	
RMET	21.22 ± 4.97	20.28 ± 6.72	.682^t^
First order false belief	2.81 ± 1.33	2.57 ± 1.13	.463^m^
Second order false belief	2.03 ± 0.89	1.14 ± 1.06	**.043** ^m^
Irony/hinting	1.51 ± 1.31	0.85 ± 0.89	.220^m^
Metaphor	1.22 ± 0.8	1.14 ± 0.37	.628^m^
Empathic understanding	2.85 ± 0.45	2.71 ± 0.48	.283^m^
Faux pas	0.25 ± 0.44	0.14 ± 0.37	.524^m^
DEToMI total score	10.00 ± 3.43	8.57 ± 3.04	.152^t^

^m^Mann–Whitney’s U-test and ^t^ T-test. Significant outcomes of P-values are reported in bold. DEToMI, Dokuz Eylül Theory of Mind Index; RMET, Reading the Mind in the Eyes Test.

In the correlation analysis between MPAs and ToM (DEToMI and RMET) performances, a negative correlation was found between mouth involvement and first-order false belief subtest performance (r=-0.381; p=0.026), and a positive correlation was found between ear involvement and irony/hinting subtest performance (r=0.364; p=0.034) in group I. Also, a negative correlation was found between mouth involvement and second-order false belief subtest performance in group II (r=-0.353; p=0.041).

Apart from these significant findings, in all groups, there was no significant association between DEToMI and RMET performances and minor physical anomalies.

### 3.5 Correlation of PANSS and theory of mind tests scores

In the correlation analysis of PANSS scores and ToM tests scores; a positive and significant correlation was found only between DEToMI ‘faux pas’ subtest and PANSS ‘Positive Symptoms’, ‘Negative Symptoms’, ‘General Psychopathology’ and total scores (r=0.488; p=0.008, r=0.379; p=0.027, r=0.425; p=0.012 and r=0.521; p=0.002, respectively) (**Table 9**).

**Table 9:** Correlation of PANSS and Theory of Mind tests scores.

	Positive symptoms		Negative symptoms		General psychopathology		PANSS total	
	r/ρ	p	r/ρ	p	r/ρ	p	r/ρ	p
RMET	-.056^r^	.753	-.290^r^	.096	-.096^r^	.590	-0.189^r^	.284
DEToMI								
1. O. F. B.	.188^ρ^	.287	.096^ρ^	.589	.061^ρ^	.730	.133^ρ^	.454
2. O. F. B.	.205^ρ^	.244	.065^ρ^	.713	.145^ρ^	.414	.195^ρ^	.270
Irony/hinting	.227^ρ^	.197	-.103^ρ^	.563	-.037^ρ^	.834	-.027^ρ^	.881
Metaphor	.036^ρ^	.841	-.263^ρ^	.132	-.247^ρ^	.159	-.168^ρ^	.341
Empathic understanding	-.089^ρ^	.618	-.156^ρ^	.379	.095^ρ^	.592	-.082^ρ^	.643
Faux pas	.488^ρ^	**.008**	.379^ρ^	**.027**	.425^ρ^	**.012**	.521^ρ^	**.002**
DEToMı Total	.197^r^	.264	-.081^r^	.649	.117^r^	.511	.087^r^	.625

^r^Pearson Correlation Coefficient and ^ρ^Spearman’s Correlation Coefficient. Significant outcomes of P-values are reported in bold. DEToMI, Dokuz Eylül Theory of Mind Index; RMET, Reading the Mind in the Eyes Test; 1. O. F. B., first-order false belief; 2. O. F. B., second-order false belief.

## 4 Discussion

In our study, only between patients and healty controls, there were significant differences in the mean scores of ‘first-order false belief’, ‘second-order false belief’, ‘faux pas’ and ‘Dokuz Eylül Theory of Mind Index (DEToMI) total’. In RMET, the mean score of patients were significantly lower than patient’s relatives and healty controls. Schizophrenia patients with mouth anomalies performed worse on the first-order false belief subtest than those without. There was a negative correlation between mouth involvement and first-order false belief subtest performance in schizophrenia patients. Additionally, schizophrenia patients with ear anomalies performed better on the irony/hinting subtest than those without, and there was a positive correlation between ear involvement and irony/hinting subtest performance. As for the patient’s relatives, those with mouth anomalies performed worse in the second order false belief subtest than those without. There was a negative correlation between mouth involvement and second-order false belief subtest performance in the patients’ relatives. Apart from these, no significant findings were found in terms of MPA, ToM performances and MPA-ToM relationship.

In the literature on the relationship between schizophrenia and MPAs, studies stating that MPAs are seen at a significantly higher rate in patients seem to predominate (14, 32–36). In a study by Griffiths et al., similar to our result, no significant difference was found between patients, their first-degree relatives and healthy controls (37). The results of MPAs studies in relatives of schizophrenic patients and healthy controls seem less consistent; it was reported that some relatives of patients had a significantly higher rate of MPAs compared to healthy controls (34, 38, 39), while in others, there was no significant difference between patient relatives and healthy controls (40–42).

The patients had the worst performance and the healthy controls had the best performance in terms of DEToMI first-order false belief, second-order false belief, faux pas subtests, DEToMI total and Eyes Test (RMET). The performance of the patients’ relatives was in between the two groups. Similar to our results, in two large meta-analysis studies on ToM in schizophrenia, patients’ ToM performances were shown to be worse than healty controls (43, 44).

It is stated that ToM has two domains: social-cognitive and social-affective (perceptual) (45). False belief tests can be used to test the social-cognitive domain, and the Eyes Test can be used to test the social-affective domain (46); faux pas is associated with both the social-cognitive and social-affective domains of ToM and is a sensitive indicator of ToM (47–50). Considering the tests in which the patient group had significantly lower scores, we found that both domains of ToM were impaired in the patient group, and this result was consistent with the literature stating that social cognition is impaired in schizophrenia. At the same time, patients scored significantly lower on the Eyes Test than the other two groups, and there was no significant difference between relatives and healthy controls on this test. Our finding of this significant difference on the Eyes Test suggests that impairment in the social-affective domain of ToM may be a disorder specific to individuals who develop schizophrenia, rather than an endophenotype indicator. Also, in the literature, the number of studies conducted by separating ToM in schizophrenia into social-affective and social-cognitive domains is few, and in studies on this subject, social-affective ToM deficit was found to be more associated with schizophrenia compared to social-cognitive ToM (51, 52).

Among patients with schizophrenia, those with mouth anomaly scored significantly lower on the DEToMI first-order false belief subtest than those without mouth anomaly, and as the number of mouth anomalies increased, first-order false belief scores decreased. In addition, in the patient relatives group, those with mouth anomalies scored significantly lower on the DEToMI second-order false belief subtest compared to those without mouth anomalies, and as the number of mouth anomalies increased, second-order false belief scores decreased. These findings were in line with our hypotheses that high MPAs rates in schizophrenic patients may be in parallel with the increased impairment in ToM, and that there may be a similar relationship between MPAs and ToM in relatives of patients. These findings may have various meanings. It is stated that each of the social-cognitive and social-affective domains of ToM are associated with certain different areas in the brain (45), and as we mentioned above, false belief tests are used to evaluate the social cognitive domain of ToM (46). Considering our finding that patients and their first-degree relatives with mouth anomaly performed significantly worse in false belief subtests, it is possible that those with schizophrenia and their first-degree relatives with mouth anomalies may be more likely to have impaired ToM performance at certain levels, at least at the social-cognitive level of ToM. In other words, we can conclude that certain early dysmorphogenetic processes (such as dysmorphogenetic processes parallel to mouth anomalies) may contribute to the level of social cognitive impairment in both patients and their first-degree relatives. As a matter of fact, mouth anomalies are the most common minor physical anomalies in schizophrenia (16) and it is stated that these anomalies are associated with pathological brain morphogenesis (53). However, those with ear anomalies in the patient group scored significantly higher in the irony/hinting subtest, and as the number of ear anomalies increased, irony/hinting scores also increased. These findings were contrary to our expectations. The finding of the present study regarding ear anomalies and irony/hinting subtest should be assessed with different samples.

All of our significant and non-significant findings regarding the MPAs-ToM relationship may be related to various factors. First of all, it is not clear whether MPAs arise genetically or due to environmental factors (54). Also, processes in morphogenesis may not always be parallel to each other. As a matter of fact, it is stated that the cortex can be reorganized and cognitive sequelae can be minimized in the later stages of the dysmorphogenetic processes that may cause MPAs (32), and the subsequent development of the brain may lead to significant differences in cognitive, neurological and clinical levels in adulthood (55). In addition, it was stated that the relationship of any of the MPAs with the dysmorphogenetic process in the brain is not clear (38). Likewise, structural/functional features of the brain can be highly affected by other factors in adulthood, such as environmental factors, healing processes, the disorder process itself, the treatment of the disorder, etc. (38). Therefore, it seems to be an assertive statement to say that the presence of an MPA and a structural/functional abnormalities in the brain completely coincide. Ultimately; MPAs and cognitive functions can be affected by many different factors (such as genetics, environmental) and it is difficult to say that there is a simple/linear relationship between them.

When we looked at the correlation between the patients’ PANSS scores and ToM test results, there was a positive correlation between the DEToMI faux pas subtest and PANSS positive symptoms, negative symptoms, general psychopathology and total scores. In the literature, studies seem to predominate showing that impairment in ToM is positively associated with negative symptoms (56–59). In a review on this subject, it was stated that in most studies, ToM impairment in patients was found to be associated with symptoms of schizophrenia (such as positive, negative and disorganized), and in a smaller number of studies, it was not associated with schizophrenia symptoms (60). In addition; Abu-Akel suggested that schizophrenia patients with positive symptoms do not have a deficiency in ToM skills, but an excess, hypertrophy (hyperToM), and that delusions basically result from excessive reference to the person’s own or others’ thoughts and intentions (61). In another study, it was stated that a deficiency in ToM was associated with negative symptoms, and an excess in ToM (hyperToM) was associated with positive symptoms (62). In the present study, only the faux pas subtest, which is considered a sensitive indicator of ToM, was positively correlated with the three main subtests of PANSS and PANSS total. Our this finding was not consistent with the literature and even contradicted most of the literature. Similar studies on ToM may contribute more to the elucidation of the relationship between schizophrenia symptoms and ToM by more comprehensive ToM performance evaluations including overmentalization.

To the best of our knowledge, the current study is the first study on the relationship between MPAs and ToM in patients with schizophrenia. Only patients and their relatives with mouth anomalies had worse first and second order false belief performances, respectively. One of the previous studies revealed associations between increased MPAs and neurocognitive dysfunctions (63). However, others did not find a relationship with MPAs and neurocognition (32, 38, 64). These inconsistent results of the studies and the low number of significant results between MPAs and ToM deficits in our study can be explained by the unclear relationship between MPAs and the dysmorphogenetic process in the brain (38), as we mentioned above. In fact, it has been stated that social cognition (an important component of which is ToM) is formed by extensive and complex network activity in the brain, rather than the activity of only a specific brain area (3). These relationships can be clarified with similar new studies on schizophrenia patients and their relatives.

Our study had several strengths and limitations. Firstly, the fact that we did not include patients according to their clinical course (for example, only first-stage patients or chronic patients) may have affected the MPA rates we determined. As a matter of fact, in Nizamie et al.’s study, higher rates of MPAs were detected in chronic schizophrenia patients compared to acute patients, but the difference was not significant (13). However, by including only patients in remission, we ensured homogeneity in the patient group in one respect. Second, we did not evaluate in detail the environmental factors that have the potential to affect both MPAs and ToM independently (for example, it is known that head circumference associated with birth complications may remain small). Third, the fact that the relatives of the patients were the attendants who came to the hospital may have affected our results, that is, it is possible that the relatives of the patients with better functionality were included in our study. Fourth, our use of the 13-item MPAS, which is less comprehensive compared to the scales that have done more comprehensive screening of a body region in most studies on similar subjects (32–36, 63, 65, 66), may have caused our low rate of MPAs. Fifth, the fact that MPA measurements were made by a single person and the findings were not supported by a second person may have caused some MPAs to be overlooked. However, the fact that only one person evaluated MPAs may also indicate that our findings are more consistent. Additionally, the fact that all other scales used in our study were administered to the participants by the same person may have prevented inconsistencies that may arise due to different testers. Sixth, it was stated that written/cartoon-based materials may not fully reflect real-life social interactions (67). Therefore, we may not have been able to evaluate ToM effectively with the written/cartoon-based materials we used to test ToM. So, it would be better to evaluate tom with more sophisticated methods. Finally; there were participants who achieved full scores on the DEToMI subtests, therefore, our results may have been affected by the ‘ceiling effect’ as highlighted by Casetta and Goghari (67). ‘ceiling effect’ is a measurement limitation that can reduce the likelihood that a test instrument will accurately test a targeted domain, which may occur when the highest possible score on the test instrument is achieved. In other words, we may not have adequately tested the ToM performances of the participants with higher levels of ToM, and we may have been insufficient to clarify the ToM performance difference between the groups. However, unlike studies that tested a single domain of theory of mind (67–69), one of the strengths of our study was that we tested both the social-cognitive and social-affective domains of the theory of mind.

As a result, MPAs may be associated with the level of social cognitive impairments in schizophrenia, so it can be argued that MPAs may have clinical importance. In addition, detailing the region in which MPAs predominate may provide information about the direction of this clinical effect. Elucidating different aspects of early maldevelopment in schizophrenia with similar studies may provide important contributions to understanding the genetics, clinical nature and treatment of the disease. It may be useful for clinicians to take into account the MPAs that may be present in schizophrenia patients in a more comprehensive evaluation of the patients’ clinics.

## Data Availability

The datasets presented in this study can be found in online repositories. The names of the repository/repositories and accession number(s) can be found in the article/supplementary material.
